# Cerebral Surgery

**Published:** 1904-07-23

**Authors:** 


*(To be continued.)*

### CEREBRAL SURGERY.

**Cerebral Tumour.**—Dr. George Woolsey[1] has collected records of 101 cases operated on within the last five years. He considers that the results obtained in this period justify a less conservative attitude toward operative treatment than at present prevails. This series shows that in many cases tumours of the prefrontal, parietal, and occipital areas can be as successfully localised and operated upon as those of the motor area. It is universally agreed that unless localising symptoms are present no operation for removal must be undertaken. Exploratory operations for suspected but non-localised tumour are nearly uniformly unsuccessful. The diagnosis therefore is of first importance. Examining the symptoms in this series we find the most constant to be headache, present in 90 per cent.; it is of no value as a localising symptom. Choked disc was present in 84·1 per cent., in 16 cases it appeared first on the same side as the tumour, in six on the opposite side. In tumours of the motor cortex it is frequently absent, *i.e.* in 33 per cent. of these cases, and when present it generally indicates a large and rapidly-growing tumour. Vomiting and vertigo were noted in 33·3 per cent. and 22·2 per cent. respectively. There are two means of localisation that may prove of much assistance; local tenderness to pressure or percussion, which was present in 21 of these cases, and *x*-rays, which were used in five; in one the result was negative, in four positive. Pfahler,[2] by means of skiagraphy, has demonstrated areas of softening in a brain in which thrombosis of the mid-cerebral artery had occurred, the softened area appearing as a light shade in the photograph. From an examination of 55 cases he believes it will be possible to diagnose tumours, softening, hæmorrhage, and abscess. Skill in technique is necessary, and familiarity with the shadows shown by the normal brain. Dr. Woolsey is in favour of operating for supposed gumma; medicinal treatment rarely if ever cures completely, moreover other tumours beside gumma may occur in syphilitic subjects, and differentiation is almost impossible for a considerable time. The mortality of cases in which the tumour was exactly localised was 22·7 per cent. as against 46 per cent. among the cases not exactly localised. The prognosis is difficult to give before operation. All cases left alone die. No doubt the operation mortality is high, but against this is to be set the certainty of death if left alone, and the relief from symptoms, especially headache, which generally ensues if only for a short time. Then, in many cases, there is a considerable period free from recurrence. Cases of sarcoma are published free from recurrence for four years, four years and one month, five and a half years, and 11 years, the last, a case of Durantes, then recurred, was operated on again, and was well six and a half years afterwards. One fibroma is reported well eight years, gummata two and a half, and two years, glioma three and a half years afterwards. Seven cases of sarcoma are reported to have recurred at intervals from three months to 11 years. Absolute cure or *restitutio in integrum* is rarely if ever to be expected on account of the necessary damage to the brain substance by the tumour and the operation of enucleating it. Still, in 16 out of the 101 cases the symtomatic result was so good as to be considered a cure and in 41 very marked improvement has followed. He recommends the palliative operation of trephining and draining to relieve pressure symptoms in cases of unlocalisable tumour. Frazier[3] also strongly advocates this measure, the distressing headache is nearly always relieved, and in some cases the changes in the optic disc have cleared up. He records five cases, and discusses the operative treatment. The improved results are, he considers, due to more accurate localisation and to improved technique, especially in more rapid operating and avoidance of hæmorrhage. These ends are to a large measure due to the method of turning down a large osteoplastic flap, and so rapidly obtaining a free exposure. Temporary closure of the carotids he considers ineffectual and not devoid of danger. He lays great stress upon the observation of blood-pressure during the operation, he has it continually recorded on a revolving drum, so that he can see at a glance the condition of the patient; if on opening the dura there is a very marked fall he postpones the rest of the operation to a later date, when the patient has recovered. If there is no great disturbance of blood pressure he sees no advantage in operating by two stages. Bulging of the brain is a troublesome feature. This occurs in two forms: (1) Initial bulging, due to increase of tension caused by the tumour; it is not always present and is not followed by (2) consecutive bulging due to traumatic œdema caused by manipulation; this is often very excessive, and suggests the absence of a tumour at that site. To deal with this bulging when the dura cannot be stitched over, he recommends the use of a periosteal flap, but only in cases where no tumour is probably present, for if a tumour be present but cannot be removed the wound should be left open and drainage established for the relief of pressure. Taylor[4] records a case of meningeal endothelioma occurring in a boy of 15, it was of very rapid growth, had invaded the skull, causing a visible external tumour, and internally symptoms of compression; at the removal there was very free hæmorrhage; the boy was relieved of symptoms and getting about well, but recurrence was noted in six weeks and he died in 10 weeks. All other cases of dural tumour that have come under his notice, with one exception, have died from hæmorrhage either at the operation or within 12 hours. Krause[5] relates four cases of non-traumatic Jacksonian epilepsy in which he operated with complete success. They comprise a cyst and a scar in the face centre, lepto-meningitis of the arm centre, and a subarachnoid cyst. In all cases the dura over the lesion appeared quite healthy and pulsating, he therefore maintains that the view of those who hold that healthy dura should not be opened is quite untenable; the cortex must be closely scrutinised, if no gross lesion can be found electrical stimulation must be used to find the centre in which the spasms begin and the centre should then be excised. The anatomical landmarks are never sufficiently definite to localise a centre without electrical stimulation. He states that the paralysis following excision of a centre generally clears up. The prognosis, as regards cure, when no gross lesion is found is not good.

[1] American Jour. Med. Sci., December 1903. [2] Ibid., February 1904. [3] Ibid. [4] Ibid. [5] Edinburgh Med. Jour., April.

